# Computed tomography versus fluoroscopic guided-sacroiliac joint injection: a prospective comparative study

**DOI:** 10.1186/s13244-021-00982-y

**Published:** 2021-03-18

**Authors:** Ahmed A. A. Bessar, Mohamed M. Arnaout, Mohammad Abd Alkhalik Basha, Shady E. Shaker, Ashraf E. Elsayed, Manar Awad Bessar

**Affiliations:** 1 Department of Radiodiagnosis, Faculty of Human Medicine, Zagazig University, Zagazig, Egypt; 2 Department of Neurosurgery, Faculty of Human Medicine, Zagazig University, Zagazig, Egypt; 3 Department of Internal Medicine, Faculty of Human Medicine, Zagazig University, Zagazig, Egypt; 4 Department of Anesthesia, Surgical Intensive Care and Pain Medicine, Faculty of Human Medicine, Zagazig University, Zagazig, Egypt

**Keywords:** Sacroiliac joint pain, Computed tomography, Fluoroscopy, Pain management

## Abstract

**Background:**

There are limited data discussing long-term pain relief and comparability of different image-guided sacroiliac joint (SIJ) injection. This study compared CT and fluoroscopic-guided SIJ injections regarding statistically and clinically significant differences in numeric pain reduction, radiation doses, and patient’s satisfaction.

**Methods:**

A prospective study conducted on 52 patients who met specific inclusion criteria of SIJ pain. A mixture of 1 ml of 40 mg methylprednisolone acetate diluted in 2 ml of lidocaine 2% was injected under either CT or fluoroscopic guidance. Numeric rating score (NRS) and Oswestry disability index (ODI) were assessed and recorded for each patient before procedure and one-week, and one-, three-, six-, and 12-months after procedure. The results were compared between both groups.

**Results:**

Analysis of NRS one-month post-procedure showed a significant decrease from baseline in both groups: 12.5% in CT group (*p* = 0.002) and 9.5% in fluoroscopic group (*p* = 0.006). No significant difference in NRS between two groups at one- and three-months post-procedure (*p* = 0.11 and 0.1, respectively). There was a significant difference in NRS between two groups at six- and 12-months post-procedure (*p* = 0.001 and < 0.0001, respectively). Comparison of ODI at six-month post-procedure revealed that both groups had a statistically significant improvement (*p* < 0.0001). There was a significant difference in ODI between two groups at six-months post-procedure (*p* = 0.01).

**Conclusions:**

CT-guided SIJ injection compares favorably with fluoroscopic guidance and offers statistically and clinically significant long-term pain relief. The use of dose reduction protocol in CT is important for decreasing the radiation dose.

## Key points

1. CT-guided SIJ injection compares favorably with fluoroscopic guidance and provide statistically and clinically significant long-term pain relief.
2. There was a significant difference in NRS between the two groups at six- and 12-months post-procedure (*p* = 0.001 and < 0.0001, respectively).
3. There was a significant difference in ODI between the two groups at six-months post-procedure (*p* = 0.01).
4. The use of dose reduction protocol in CT is important for decreasing the radiation dose.
5. High percentage of patients in CT group were strongly satisfied with the procedure and results compared to those in fluoroscopic group.

## Background

The sacroiliac joint (SIJ) is a weight-bearing, diarthrodial joint that consists of a fibrous capsule and synovium. The superior part of the joint is ligamentous, while the inferior part is articular cartilage [1]. SIJ pain is a common etiology of low back pain (LBP) and has been reported to be a source of pain in 15 to 30% of patients with chronic LBP [1, 2]. Medical history and physical examination are not adequate to reliably diagnose those with SIJ pain [3, 4]. The patient’s response to an image-guided intra-articular injection with an anesthetic is considered the current clinical standard for diagnosing SIJ pain [1, 3, 5].

The SIJ has a complex and variable anatomy, such that local treatment can be a therapeutic and technical challenge [6, 7]. Imaging guidance is considered the gold standard for SIJ injection [8]. Fluoroscopy, computed tomography (CT), ultrasound (US), and magnetic resonant imaging (MRI) have been recommended for image-guided procedures. Each imaging modality has an institutional preference in routine practice according to physicians’ experiences and backgrounds [9–13].

The efficacy of different imaging methods to guide SIJ injections has been extensively discussed, resulting in very high success rates of up to 90% [14–18]. However, data discussing the long-term pain relief and the comparability of different image-guided techniques are limited. To report the gaps in current knowledge about the efficacy of different image-guided methods, we conducted this prospective study comparing the therapeutic outcome of CT-guided SIJ injection to that of fluoroscopic guidance.

## Methods

### Study design

A prospective cohort study was conducted in a group of patients with SIJ pain seeking pain relief by SIJ injection.

### Ethical considerations

The Institutional Review Board approved the study. The risk and potential benefits of the procedures were explained to all patients, and written informed consent was obtained before participating in the study. The study followed the ethical principles of the Declaration of Helsinki.

### Study population

**Fig. 1:**
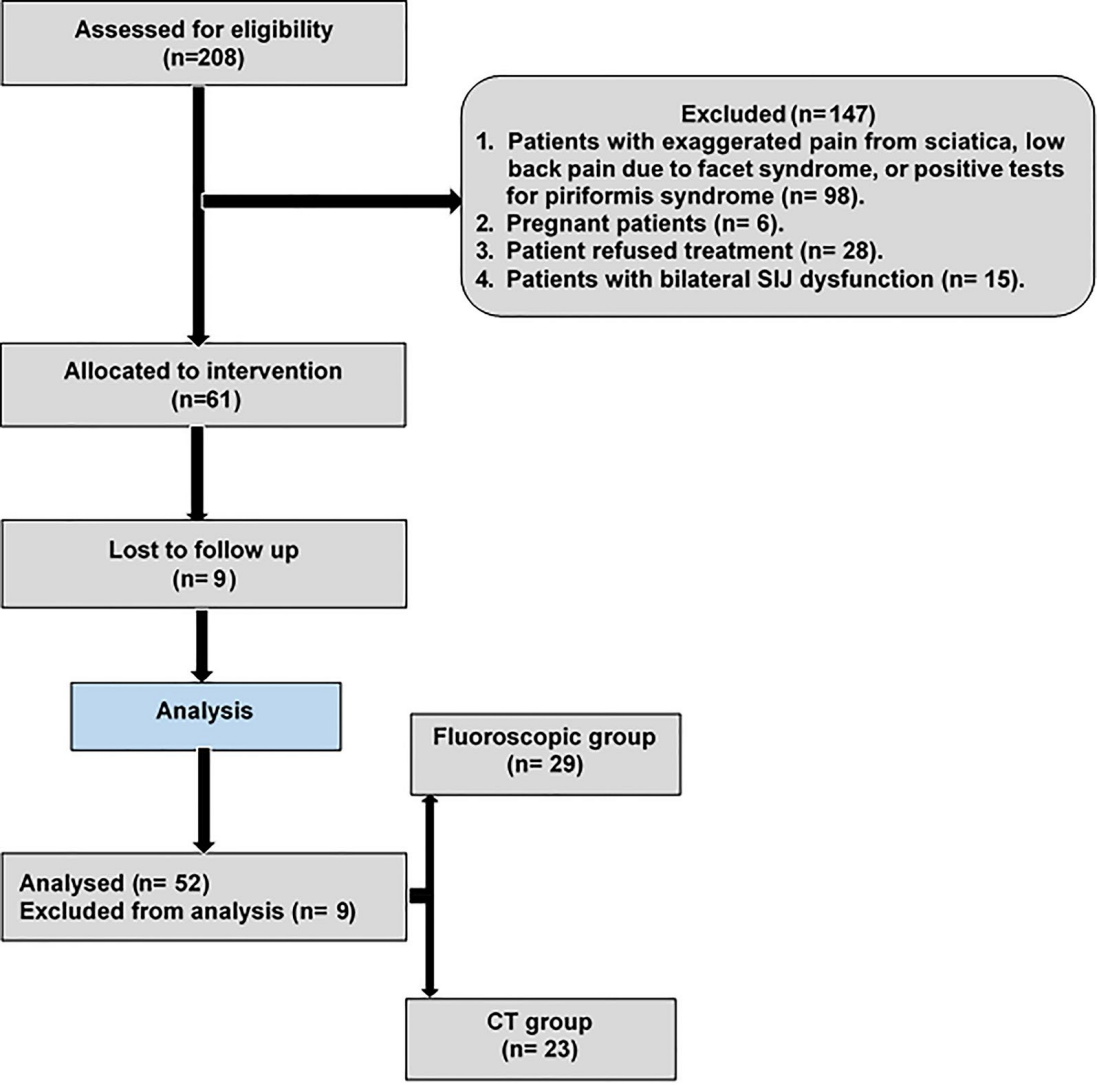
Flowchart of our study

The study was carried out in a tertiary health institution between January 2017 and February 2020. Initially, a total of 208 patients with chronic LBP were screened for SIJ injections. The inclusion criteria were (i) patients who had functionally limiting pain that originated from the SIJ and rated 4 or higher on the 0–10 numeric rating score (NRS), (ii) patients more than 18 years old, (iii) patients who had failed conservative care that consisted of physical therapy or medications, and (iv) patients with failed medical therapy. Exclusion criteria were (i) patients with exaggerated pain from sciatica, and low back pain due to facet syndrome, or positive tests for piriformis syndrome (*n* = 98), (ii) pregnant patients (*n* = 6), (iii) patients refused treatment (*n* = 28), (iv) patients with bilateral SIJ dysfunction (*n* = 15). A total of 61 patients met the diagnostic criteria for SIJ pain and were eligible to participate in the study. Nine patients were lost to follow-up early during the first 6-month of the study and excluded from the final analysis. Fifty-two patients were included in the final analysis. The patients were randomly categorized into two groups: fluoroscopic group, included 29 patients who underwent fluoroscopic-guided SIJ injection, and the CT group included 23 patients who underwent CT-guided SIJ injection. Figure 1 illustrates the flow diagram of our study.

### Patient assessment

Before injection, all patients were interviewed and subjected to clinical examination. All clinical maneuvers were performed by an independent physician, including the abduction, flexion, external rotation, sacral distraction, Gaenslen’s test, sacral thrust, thigh thrust, and lateral compression. All prior radiological procedures, including previous plain radiographic examinations and MRI images, were reviewed. At presentation and before image-guided injection, a baseline NRS and Oswestry disability index (ODI) were calculated and registered for each patient. The ODI was scored out of 50 points and converted to a percentage, with higher scores indicating greater disability.

### Procedure

All injections were performed in an outpatient basis by one interventional radiologist (A.A.A.B., with 10 years of experience). Injections were done unilaterally according to clinical examinations. Patients were randomized to either CT-or fluoroscopic-guided injections by one of the authors using serially numbered containers. All procedures were performed with patients in a prone position, and the area of injection was exposed. Neither patient required sedation nor preoperative pain medication. The injection was done at the lowermost inferior part of the joint. The mixture used for injection was 1 mL of 40 mg of methylprednisolone acetate (Depo-Medrol; Pfizer Canada Inc., Kirkland, Quebec, Canada) diluted in 1 mL of 2% lidocaine. After each injection, the puncture tract was flushed with 0.5 mL lidocaine 2% while retrieving the needle to avoid spread of corticoid medication.

#### Fluoroscopic-guided injection

**Fig. 2:**
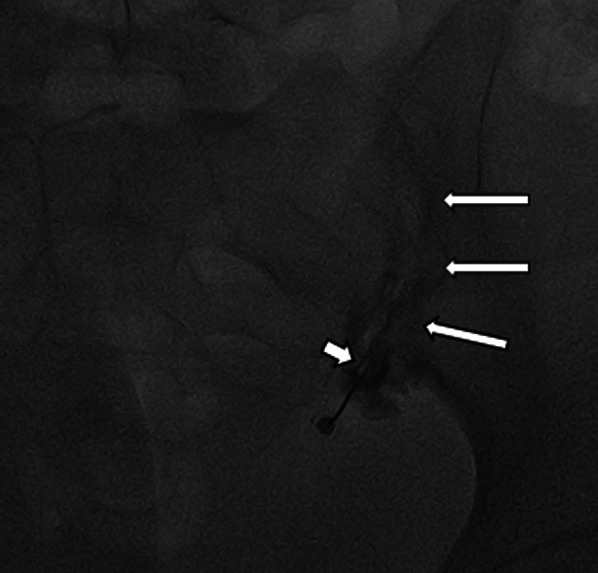
A 60-year-old lady with sacroiliitis referred for SIJ injection. Fluoroscopic image obtained during SIJ injection shows the insertion of the needle tip (short arrow) within the inferior aspect of SIJ, and the linear contrast pattern (long arrows) along the course of SIJ extends away from the needle tip. Note; some extra-articular spread of contrast away from the needle tip

All fluoroscopic-guided injections were performed using Artis zee C-arm (Siemens healthcare). The SIJ was detected by moving the tube to the contralateral oblique view for 15°–45° and slightly cephalad for 10°–20°. This angulation made the lines of both anterior and posterior aspects of the joint overlapped, and the joint became visualized in profile view. After localization of the needle tip in the distal part of the posterior aspect of the joint and ensuring sterilization of the exposed area, a local anesthetic (1 mL of lidocaine 2%) was injected. A 22G Quincke spinal needle, 10-cm-long, was inserted under continuous fluoroscopic guidance until the proper position in the joint space. A maximum of 0.5 mL of non-ionic contrast “Iohexol” (omnipaque™, GE healthcare) was injected to ensure the proper position of the needle tip that was confirmed by the upward spread of the contrast to the uppermost parts of the joint (Fig. 2).

#### CT-guided injection

**Fig. 3:**
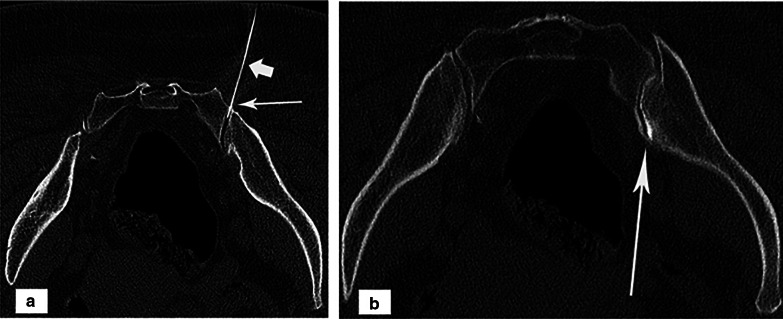
A 50-year-old lady with sacroiliitis referred for SIJ injection. **a** A non-enhanced CT (bone window) lower cut on SIJ shows the synovial part of the joint (long arrow) where the needle (short arrow) is inserted. **b** A contrast-enhanced CT (bone window) upper cut reveals contrast (long arrow) within the anterior synovial part of the joint. No contrast in the posterior fibrous part of the joint

All scans were performed using a 128-multidetector CT scanner (Ingenuity, Philips healthcare) with collimation of 128 × 0.625, slice thickness, and interval of 4 mm, a pitch of 0.993, a display field of view of 42 cm^2^, a matrix of 512 × 512, a gantry rotation of 330 ms. A biopsy mode with reduced energy and tube current (80 kV, 50 mAs) was used. Initial scans were done, and the preferred location for injection was marked by a laser beam on the surface of the back. Each joint needed an average of 3 to 5 scans for proper needle localization. A local anesthetic (1 mL of lidocaine 2%) was injected into the chosen point of injection after sterilization. A 22G Quincke spinal needle, 10-cm-long, was inserted toward the synovial part of the joint under serial cuts of CT (mean, 5.3 ± 1.7; range, 3–10). A maximum of 0.5 mL of non-ionic contrast “Iohexol” (omnipaque™, GE healthcare) was injected to ensure the proper position of the needle tip. After placement of the needle tip into the joint space, the mixture was injected (Fig. 3).

### Post-procedure

Immediate post-procedural pain relief should be experienced in all patients to be enrolled in the study also, to confirm the absence of other sources of pain.

All patients were asked to get rest in the recovery area for two hours before going home. No postoperative oral pain medications were prescribed to monitor the degree of pain relief and prevent any bias for pain evaluation.

### Follow-up

All patients were followed-up regularly, with periodical clinical visits at one week and 1, 3, 6, and 12 months after the procedure. The same physician who performed the initial clinical assessment also performed the re-examination with the same clinical maneuvers. The postoperative follow-up included a clinical examination of SIJ pain and its degree of relief, NSR, and ODI. The NRS and ODI values were reported at each visit and compared with the baseline values. The percent reduction was calculated using the formula (pre-procedure—post-procedure)/pre-procedure × 100. The total radiation dose was calculated automatically for each patient. The total duration of the procedure from area exposure until the end of the procedure was calculated for each patient. Patient satisfaction for the procedure was assessed on the day of the procedure, and patient satisfaction for the results of the procedure was evaluated after six months. Patient satisfaction was nominated in terms of strongly satisfied, mildly satisfied, and not satisfied according to the patients' own words and expressions.

### Statistical analysis

The data obtained were analyzed using MedCalc version 11.1. The means and standard deviations were calculated for quantitative data, and numbers and percentages were calculated for categorical data. Pre- and post-procedure results were compared using a paired sample *t*-test and Mann–Whitney *U*-test when appropriate. Categorical variables were analyzed using the Chi-square test or Fisher’s exact test. A *p* value < 0.05 was accepted as significant.

## Results

### Patients

A total of 52 patients with SIJ pain (29 males and 23 females; mean age, 46 ± 12.1 years; range, 30–67 years) were included in the final analysis. The basic demographic data of the patients are described in Table 1. No significant differences between the two groups in terms of pre-procedure NRS, limitation of physical function as measured by ODI, baseline characteristics, or the duration of pain before the procedure. Right-sided SIJ injection was performed in 28 patients, and left-sided injection was performed in 24 patients. All procedures were successfully completed without any immediate or delayed complications. In the fluoroscopic group, there was difficult localization of the joint space in five patients with advanced osteophytosis, three patients with complex anatomy, and two obese patients. In those patients, we increased the amount of contrast to access the joint.

**Table 1:** Baseline patients’ data

	All patients (*n* = 52)	Fluoroscopic group (*n* = 29)	CT group (*n* = 23)	*P* value
Age	46 ± 12.1 (30–67)	44.4 ± 11.3 (32–60)	48.2 ± 13.9 (30–67)	0.56
Sex, *n* (%)				0.79
Male	29 (55.8)	15 (51.7)	14 (60.9)	
Female	23 (44.2)	14 (48.3)	9 (39.1)	
BMI (kg/m^2^)	26.9 ± 7.5 (20.2–34.8)	27.3 ± 8.2 (21.5–33.1)	25.9 ± 6.5 (20.2–34.8)	0.58
Side of pain, *n* (%)				0.57
Right	28 (53.8)	17 (58.6)	11 (47.8)	
Left	24 (46.2)	12 (41.4)	12 (52.2)	
Duration of pain (years)	4.9 ± 3.5 (1–12)	5.4 ± 3.7 (2–12)	4.3 ± 3.4 (1–10)	0.61
NRS	7.3 ± 0.94 (5–9)	7.4 ± 0.91 (6–9)	7.2 ± 0.98 (5–9)	0.45
ODI	59.5 ± 16.1 (30–90)	59.0 ± 17.0 (30–90)	60.2 ± 15.2 (35–85)	0.79

Unless otherwise indicated, data are mean ± standard deviation and data in parentheses are range. CT = computed tomography; NRS = Numerical Rating Score; ODI = Oswestry Disability Index

### Comparison of clinical outcomes between the two groups

Table 2 compared between both groups regarding outcome measures. Analysis of NRS one-month post-procedure showed a significant decrease from the baseline in both groups: 12.5% in the CT group (*p* = 0.002) and 9.5% in the fluoroscopic group (*p* = 0.006). Although there was a statistically significant improvement in both groups, there was no clinically significant difference in NRS between the two groups at one- and 3-months post-procedure (*p* = 0.11 and 0.1, respectively). There was a statistically significant difference in NRS between the two groups at six- and 12-months post-procedure (*p* = 0.001 and < 0.0001, respectively). Comparison of ODI at six-month post-procedure revealed that both groups had a statistically significant improvement (reduction by 51.3% and 35.4%, respectively, *p* < 0.0001). There was a significant difference in ODI between the two groups at six-months post-procedure (*p* = 0.01). The number of patients with ODI below 20 (minimal disability) was 5 in the CT group, whereas none in the fluoroscopic group had ODI below 20. The number of patients with ODI between 21 and 40 (moderate disability) was 16 in the CT group and 13 in the fluoroscopic group. Regarding the total duration of the procedure, there was no significant difference between the two groups (13.8 vs. 14.1 min, *p* = 0.5). Regarding the radiation doses, no significant differences were observed between the two groups (13.2 vs. 14.5 mGycm^2^, *p* = 0.09).

**Table 2:** Comparison of outcome measures between the two groups

	Fluoroscopic group (*n* = 29)	CT group (*n* = 23)	*P* value
NRS			
Pre-procedure	7.4 ± 0.91 (6–9)	7.2 ± 0.98 (5–9)	0.45
Post-procedure 1 week	7.2 ± 0.86 (5–8)	6.7 ± 0.9 (5–8)	0.98
Post-procedure 1 month	6.7 ± 0.94 (5–8)	6.3 ± 0.82 (5–7)	0.11
Post-procedure 3 months	5.6 ± 0.49 (5–6)	5.3 ± 0.82 (4–7)	0.1
Post-procedure 6 months	4.3 ± 0.89 (3–6)	3.5 ± 0.73 (2–5)	0.001
Post-procedure 12 months	2.8 ± 0.99 (1–4)	1.6 ± 0.65 (1–3)	< 0.0001
ODI			
Pre-procedure	59.0 ± 17.0 (30–90)	60.2 ± 15.2 (35–85)	0.79
Post-procedure 6 months	38.1 ± 12.7 (20–75)	29.3 ± 11.2 (15–60)	0.01
Duration of the procedure (minutes)	14.1 ± 3.2 (10–20)	13.8 ± 5.4 (10–24)	0.5
Total radiation dose (mGycm^2^)	13.2 + 2 (6.7–16)	14.5 ± 2.5 (13–25)	0.09

Data are mean ± standard deviation. Data in parentheses are range. CT = computed tomography; NRS = Numerical Rating Score; ODI = Oswestry Disability Index

### Comparison of overall patient satisfaction between both groups

Eighteen (78.3%) patients were strongly satisfied with the CT procedure compared to 10 (34.5%) patients in the fluoroscopic group. Also, 14 (60.9%) patients were strongly satisfied with the CT results compared to 4 (13.8%) patients in the fluoroscopic group (Table 3).

**Table 3:** Overall patient satisfaction

	Fluoroscopic group (*n* = 29)	CT group (*n* = 23)	*P* value
For procedure			0.004
Strongly satisfied	10 (34.5)	18 (78.3)	
Mildly satisfied	19 (65.5)	5 (21.7)	
Not satisfied	0	0	
For the result			0.0004
Strongly satisfied	4 (13.8)	14 (60.9)	
Mildly satisfied	15 (51.7)	2 (8.7)	
Not satisfied	10 (34.5)	7 (30.4)	

Data are number of patients. Data in parentheses are percentages. CT = computed tomography

## Discussion

The main objective of any image-guided techniques for SIJ injection is to relieve pain rapidly and safely. In this study, we examined two image-guided techniques—the CT and fluoroscopic-guided injection. Although the efficacy of these techniques and their outcomes has been excessively investigated [19–24], up to date, the comparison between CT and fluoroscopy-guided SIJ injection has not been tested. The present study prospectively compared CT-guided SIJ injection and fluoroscopic guidance regarding the statistically and clinically significant differences in numeric pain reduction.

When an accurate diagnosis is of high significance, the accuracy of the injection is crucial. However, in clinical practice, the final therapeutic outcome may be more significant than the accuracy of the injection [25]. In comparing CT to fluoroscopically guided SIJ injection, our study found a statistically significant improvement with no clinically significant difference in NRS at one-week and one- and three-months post-procedure (*p* = 0.98, 0.11, and 0.1, respectively). However, at six- and 12-months, there was a statistically significant difference in NRS between two groups (*p* = 0.001 and < 0.0001, respectively). Also, at six months, a statistically significant difference was observed in ODI between the two groups (*p* = 0.01).

The number of patients with ODI below 20 (minimal disability) was 5 in the CT group, whereas none in the fluoroscopic group had ODI below 20. The number of patients with ODI between 21 and 40 (moderate disability) was 16 in the CT group and 13 in the fluoroscopic group. Therefore, the CT-guided injection provided a long-term pain reduction compared to fluoroscopic guidance. These findings support the previous literature [22, 23], which has reported long-lasting effects of CT-guided injections in patients with SIJ pain. Also, Dussault et al. [9] found that 97% of fluoroscopically guided SIJ injections were intra-articular. However, it is difficult in some cases to place the needle into the joint under fluoroscopic guidance. In such cases, CT guidance can facilitate proper needle placement.

Based on our findings, which confirm those of previously published studies [22–24] and in view of the significant performance of CT-guided injection on prolonged SIJ pain reduction, our data support that CT-guided injection is potentially slightly better with long-term pain relief of SIJ pain than fluoroscopic guidance. The long-term pain relief by CT-guided injection in our study could be attributed to the injection of all mixtures in the intra-articular space. In contrast, there was a loss of some of the mixture during injection under fluoroscopic guidance due to difficult localization of the joint space in patients with advanced osteophytosis (n = 5) or obese patients (*n* = 2), or due to the complexity of the anatomy (*n* = 3).

Stoeckelhuber et al. [26] stated that although the fluoroscopy and CT are regarded as well-established methods to monitor the interventional treatment of SIJ pain, their radiation exposure is a disadvantage to both the interventional radiologist and younger patients. Also, Artner et al. [27] reported that despite accurate intra-articular injection in CT-guidance, radiation exposure persists as a crucial problem. Shepherd et al. [28] reported general radiation doses of 199 mGycm^2^, and Schmid et al. [29] estimated average radiation doses of 100.7 to 235.3 mGycm^2^ for conventional CT-guided spinal interventions. In our study, no significant difference was observed between the two groups regarding radiation dose (*p* = 0.09) because we used the dose reduction protocol in CT-guided injection with proper needle position in the scout series to limit the planning axial images. The application of dose reduction protocols that diminished radiation exposure by more than 90% has been reported in a study performed by Artner et al. [27], which achieved a mean radiation dose of 4.6 mGycm^2^ for CT-guided SIJ injection.

Our study revealed that 78.3% of patients in CT group were strongly satisfied with the procedure compared to 34.5% of patients in fluoroscopic group (*p* = 0.004). As well as, 60.9% of patients in CT group were strongly satisfied with the result of the procedure compared to 13.8% of patients in fluoroscopic group (*p* = 0.0004).

This study had limitations. First, small sample size in each arm of the study. Second, the effect of extra-articular or combined intra- and extra-articular injections to control pain form SIJ dysfunction in short- and long-term follow-up visits were not performed. Third, no comparison regarding the accuracy of each procedure. Fourth, the exclusion criteria were very strict and may limit the generalizability of the results to many practices. So further studies comparing both techniques in all patients are recommended. Fifth, the multifactorial nature of LBP with a significant proportion of pain from arthropathies in facet joints at the above levels, radiculopathy or spinal instability. Sixth, the lack of pre-procedural provocative test to measure the potential response to either fluoroscopic or CT-guided injection. Seventh, the subjective way performed in most cases to measure the degree of pain relief according to patient’s words. Finally, the extra-articular spread of injectate in fluoroscopic guided interventions and the inability to localize the needles properly in some patients under X-ray guidance could be disadvantages.

## Conclusions

CT-guided SIJ injection compares favorably with fluoroscopic guidance and offers statistically and clinically significant long-term pain relief. Both techniques have similar radiation doses. However, the use of dose reduction protocols in CT is important for decreasing the radiation dose. The CT-guided injection can become a widely accepted procedure for the treatment of SIJ pain. Further studies are still required to establish the long-lasting efficacy and safety of CT-guided SIJ injection compared to other image-guided techniques.

## Data Availability

The datasets used and/or analyzed during the current study are available from the corresponding author on reasonable request.

## Abbreviations

CT: Computed tomography
LBP: Low back pain
NRS: Numeric rating score
ODI: Oswestry disability index
SIJ: Sacroiliac joint
